# Wildlife Hosts of *Leishmania infantum* in a Re-Emerging Focus of Human Leishmaniasis, in Emilia-Romagna, Northeast Italy

**DOI:** 10.3390/pathogens11111308

**Published:** 2022-11-07

**Authors:** Roberta Taddei, Arianna Bregoli, Giorgio Galletti, Elena Carra, Laura Fiorentini, Maria Cristina Fontana, Matteo Frasnelli, Carmela Musto, Giovanni Pupillo, Alessandro Reggiani, Annalisa Santi, Arianna Rossi, Marco Tamba, Mattia Calzolari, Gianluca Rugna

**Affiliations:** 1 Istituto Zooprofilattico Sperimentale della Lombardia e dell’Emilia Romagna, via A. Bianchi 9, 25124 Brescia, Italy; 2 Department of Veterinary Medical Sciences, University of Bologna, Via Tolara di Sopra 50, 40064 Ozzano Emilia, Italy

**Keywords:** *Leishmania infantum*, reservoir, wildlife, artiodactyls, roe deer, European hare, red fox, wild boar

## Abstract

In the last decade, an upsurge of human leishmaniasis has been reported in the Emilia-Romagna region, Northeast Italy. Epidemiologic data have raised doubts about the role of dogs as the main reservoirs for *Leishmania infantum*. In the present study, a total of 1077 wild animals were screened for *L. infantum* DNA in earlobe and spleen samples from 2019 to 2022. The lymph nodes were tested only in 23 animals already positive in the earlobe and/or spleen. A total of 71 (6.6%) animals resulted positive in at least one of the sampled tissues, including 3/18 (16.7%) wolves, 6/39 (15.4%) European hares, 38/309 (12.3%) roe deer, 1/11 (9.1%) red deer, 8/146 (4.9%) wild boars, 13/319 (4.1%) red foxes, 1/54 (1.9%) porcupine, and 1/59 (1.7%) European badger. Most of the infected animals (62/71) tested positive only in the earlobe tissue, only four animals (two roe deer and two wild boars) tested positive only in the spleen, and five animals (three roe deer and two red foxes) resulted positive for both tissues. *L. infantum* DNA was detected in the lymph nodes of 6/23 animals. *L. infantum* detection occurred in all seasons associated with low real-time PCR Ct values. Further research is needed in order to clarify the role of wildlife in the re-emerging focus of leishmaniasis in Northeast Italy.

## 1. Introduction

Leishmaniasis caused by the protozoan *Leishmania* (*L*.) *infantum* Nicoll, 1908 is a neglected vector-borne zoonotic disease that is endemic in the Mediterranean basin [1,2]. *L. infantum* is the causative agent of zoonotic visceral (VL), cutaneous (CL) and mucosal (ML) leishmaniasis in humans [3,4]. It is also the cause of canine leishmaniasis (CanL) in dogs, which are considered the major domestic reservoir of infection for people [5]. In the Old World, transmission occurs via the bite of female sand flies of the genus *Phlebotomus* [1].

*L. infantum* is endemic in Italy, as it is throughout the entire Mediterranean region, with a median CanL seroprevalence of 17.7% [6]. The classical endemic areas are the rural and hilly peri-urban zones along the Tyrrhenian littoral, the southern peninsular regions, and the islands. Since the 1990s, the incidence of leishmaniasis in both dogs and humans has been on the rise in northern areas, previously regarded as non-endemic [7]. Among the northern Italian regions, the Emilia-Romagna region (RER) is characterized by a distinctive epidemiological situation: since 1934, CL has been frequently reported—with an incidence exceeding 2000 cases in the 1950s—before declining, most likely as a result of pesticide use in agriculture [8]. In contrast, until the early 1970s, there were only four recorded autochthonous cases of VL. In 1971–1972, a severe VL outbreak with 60 cases occurred in the foothill areas close to Bologna municipality, which is located in the central RER [9]. This outbreak was regarded as atypical, as only human cases were diagnosed, but a canine reservoir was not clearly evidenced [9]. Furthermore, follow-up surveillance of this VL focus failed to reveal human and canine cases over the next 15 years [10].

Since 2012, an upsurge in VL cases has been described in RER [11,12]. Similar to the VL outbreak in the 1970s, no rise in *L. infantum* infection among dogs was reported, which motivated research to better understand the regional *L. infantum* epidemiology. Molecular typing studies showed that *L. infantum* strains circulating in dogs belonged to a distinct population in comparison with strains circulating in VL cases and sand flies [13,14]. Moreover, the blood-meal analysis of *Phlebotomus* (*Ph*). *perfiliewi* Parrot, 1930—the strongly suspected vector of *L. infantum* in RER [15]—showed a biting preference for wild animals and humans [16]. These findings led to hypothesize a role for an unknown wildlife species as a reservoir for the parasite, also according to the increasing number of studies reporting infection of *Leishmania* spp. in wild animals [17]. 

The aim of the present study was to evaluate the role of different wild mammals as natural hosts of *L. infantum* in RER by testing them for *L. infantum* DNA over a 3-year period.

## 2. Materials and Methods

### 2.1. Study Area

RER is located in the northeastern part of the Italian peninsula. It covers an area of approximately 22,500 km^2^ and is inhabited by a population of 4.5 million people. The region is geographically divided into two homogeneous areas: the northern half of the region is entirely occupied by the Po Valley, while in the southern half, the flat area gives way to the hilly and then to the mountainous part of the Apennine chain. On its eastern side, it borders the Adriatic Sea. The climate is sub-continental in the inner part of the region and becomes Mediterranean near the coasts. The average temperature from 1991 to 2015 was 12.8 °C. The rainfall ranges from 650 to 1200 mm per year [18], depending on the altitude and the distance from the sea: the minimum value is recorded in the plains and increases toward the hills and the mountains. The rains are concentrated mainly in autumn and spring; the summer is often characterized by severe dryness. 

RER is divided into 9 administrative provinces; this study included the warm-blooded fauna of the 6 provinces most affected by human leishmaniasis in the last decade: Reggio Emilia, Modena, Bologna, Ravenna, Forlì-Cesena, and Rimini [19]. This territory comprises the central-eastern part of the region, characterized by flat, hilly, and mountainous areas as well as the Adriatic coast.

### 2.2. Sampling

From February 2019 to March 2022, a total of 1077 wild mammal carcasses were collected as part of the wildlife health surveillance program set up in RER [20].

They consisted of: 508 artiodactyls, including 309 roe deer (*Capreolus capreolus* Linnaeus, 1758), 164 wild boars (*Sus scrofa* Linnaeus, 1758), 24 fallow deer (*Dama dama* Linnaeus, 1758), 11 red deer (*Cervus elaphus* Linnaeus, 1758); 401 carnivores, including 319 red foxes (*Vulpes vulpes* Linnaeus, 1758), 59 European badgers (*Meles meles* Linnaeus, 1758), 18 wolves (*Canis lupus italicus* Altobello, 1921), 2 stone martens (*Martes foina* Erxleben, 1777), 1 pine marten (*Martes martes* Linnaeus, 1758), 1 raccoon (*Procyon lotor* Linnaeus, 1758), 1 Western polecat (*Mustela putorius* Linnaeus, 1758); 67 rodents, including 54 porcupines (*Hystrix cristata* Linnaeus, 1758), 7 squirrels (*Sciurus vulgaris* Linnaeus, 1758), 5 dormices (*Glis glis* Linnaeus, 1766), 1 common dormouse (*Muscardinus avellanarius* Linnaeus, 1758); 62 hedgehogs (*Erinaceus europaeus* Linnaeus, 1758) belonging to the Eulipotyphla order; 39 lagomorphs European hares (*Lepus europaeus* Pallas, 1778).

The animals were legally hunted, found dead by local authorities, accidentally road-killed, or conferred by wildlife rescue and rehabilitation centers.

Postmortem examination and tissue sampling were conducted at the Istituto Zooprofilattico Sperimentale della Lombardia e dell’Emilia-Romagna. The presence of pathological alterations in the organs was recorded. Spleen and earlobe samples were collected from all animals included in the study. Superficial lymph nodes of the head were sampled in a limited number of carcasses and tested only if the spleen and/or earlobe resulted positive.

All the sampled tissues were stored frozen (−20 °C) until molecular analysis.

### 2.3. Molecular Analysis

Genomic DNA was extracted from 25 mg sample of the spleen, lymph node, and the central hairless part of the earlobe. Tissue samples were homogenized and incubated overnight at 56 °C in 200 μL buffered lysis solution containing proteinase K (10 μg/mL). DNA was extracted using the NucleoSpin Tissue Kit (Macherey Nagel, Duren, Germany) according to the manufacturer’s instructions and eluted in 200 μL of elution buffer. Each sample was co-extracted and amplified with a commercial internal control DNA template (QuantiFast Pathogen PCR +IC Kit, Qiagen GmbH, Hilden, Germany). The detection of *Leishmania* DNA was carried out by a TaqMan MGB probe real-time PCR targeting a highly repetitive kinetoplast minicircle DNA sequence [21]. Each amplification was performed in a 25 μL of reaction mixture which contained 1x QuantiFast Pathogen Master Mix, 1x Internal Control Assay, 300 nM of each primers (leish-F: 5′-ACTTTTCTGGTCCTCCGGGTAG-3′, leish-R: 5′ -CCTATTTTACACCAACCCCCAGT-3′) 200 nM of probe (Leish Pb FAM-ATTTCTGCACCCATTTT-MGB), and 5 μL of sample DNA. The reactions were carried out on BioRad CFX96 thermal cycler (Bio-Rad Laboratories, Hercules, CA, USA) with the following temperature profile: 5 min at 95 °C, then 45 cycles of 15 s at 95 °C and 30 s at 60 °C. The PCR was considered positive when a threshold cycle (Ct) value lower than 40 was detected.

### 2.4. Data Analysis

Data were summarized with the proportion of positive samples, with exact confidence intervals [22]. Analysis was performed with R 4.0.2 [23]. The spatial distribution of samples was provided with QGis (version 3.14.15-Pi).

## 3. Results

A total of 2177 tissue samples were analyzed for the presence of *L. infantum* DNA: spleen and earlobe samples from each of the 1077 animals included in the study, and the lymph nodes of 23 animals found positive in the earlobe and/or spleen. 

The overall species and tissue-specific PCR detection is summarized in Table 1. A total of 71 (6.6%) animals resulted positive in at least one of the sampled tissues, including 3/18 (16.7%) wolves, 6/39 (15.4%) European hares, 38/309 (12.3%) roe deer, 1/11 (9.1%) red deer, 8/164 (4.9%) wild boars, 13/319 (4.1%) red foxes, 1/54 (1.9%) porcupine, and 1/59 (1.7%) European badger.

In relation to the tissue location of the parasite, five animals (three roe deer and two foxes) tested positive in both the spleen and earlobe, four animals (two roe deer and two wild boars) tested positive only in the spleen, while the large majority of the positive animals (62/71) tested positive only in the earlobe. Earlobe samples showed the highest *L. infantum* DNA detection rate (6.2%; CI 95%: 4.9–7.8) compared to spleen samples (0.8%; CI 95%: 0.4–1.6). The superficial lymph nodes were tested on a subgroup of 23 animals already positive for the ear (19), spleen (1), or both (3). Among them, six animals tested positive, and one roe deer resulted positive in all the three organs (earlobe, spleen and lymph nodes) (Table 2).

None of the positive animals showed macroscopic dermal and/or internal lesions suggestive of *Leishmania* infection, with the exception of one roe deer with a localized ulcerative lesion located on the positive earlobe (further investigations are underway).

**Figure 1:**
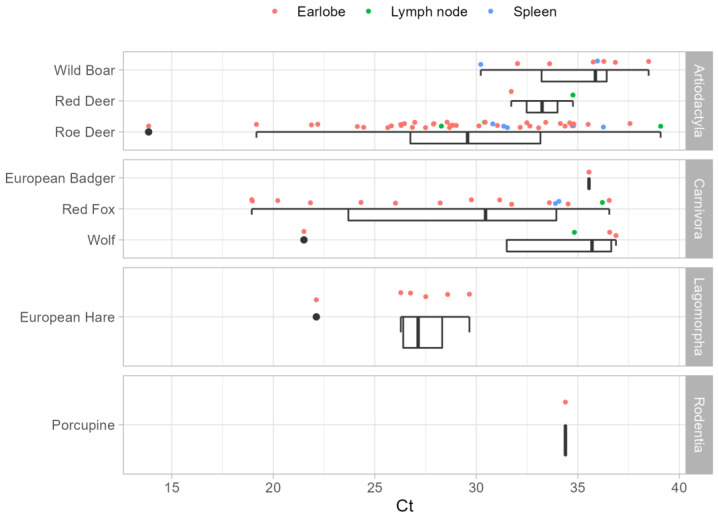
Distribution of *L. infantum* real-time PCR cycle threshold (Ct) values for the target species and tissues.

The PCR Ct values ranged from 13.9 to 39.1; 15.9% of the samples had Ct values lower than 25, and 17.1% of the samples had Ct values greater than 35 (Figure 1). Overall, median Ct values were lower in the earlobe than in spleen and lymph node samples, with results of 29.0, 33.9, and 34.8, respectively. In particular, the median Ct values were less than 35 only in the earlobes of roe deer, red deer, red foxes, European hares, and porcupines. In addition, Ct values resulted less than 20 in four earlobe samples (two from roe deer and two from red foxes), always exceeding 30 in all spleen samples.

**Figure 2:**
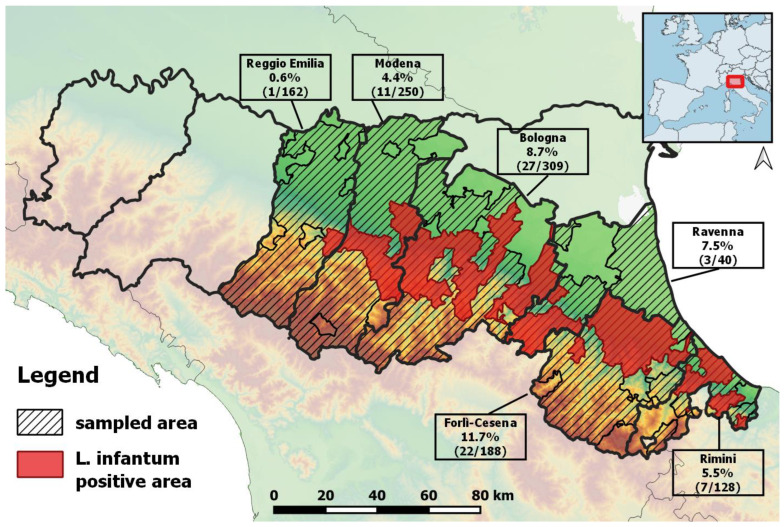
Sampled and *L. infantum* real-time PCR positive areas, Emilia-Romagna region, Italy.

The spatial distribution of both the sampled and positive animals is shown in Figure 2. The highest detection rates were concentrated in the provinces of Forlì-Cesena (11.7%) and Bologna (8.7%) (Figure 2 and Table 3). At the province level, the highest number of positive species was also recorded in the Bologna and Forlì-Cesena provinces, with five and four species, respectively (Table S1).

**Figure 3:**
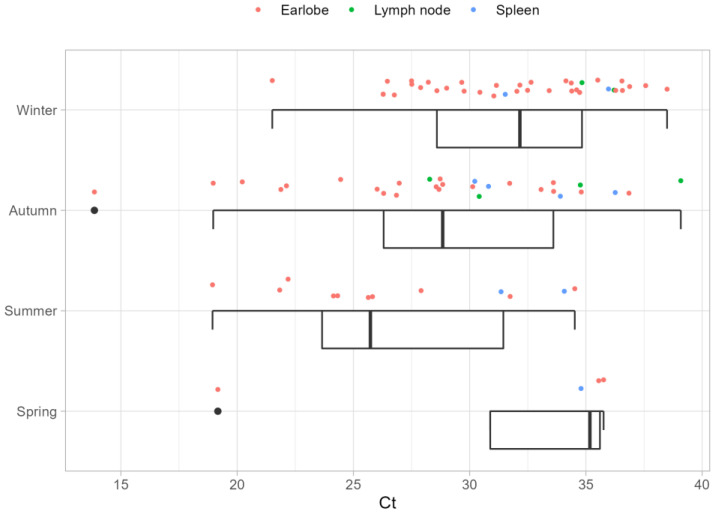
Distribution of *L. infantum* real-time PCR cycle threshold (Ct) values among the weather seasons.

The yearly temporal distribution of *Leishmania* DNA detection showed that the animals tested positive in all seasons, and Ct values lower than 25 (from 13.9 to 21.6) were recorded throughout the whole year (Figure 3). Roe deer tested positive in all seasons (Table 4), with minimum associated Ct values ranging from 13.9 to 26.3. Overall, one roe deer, positive in both the earlobe and spleen, one badger, and one wild boar tested positive for *L. infantum* DNA during spring (Table 4).

## 4. Discussion

*L. infantum* is a zoonotic parasite steadily endemic at a low prevalence (around 2%) in the canine population in RER [19,24]. In contrast, an increasing number of VL, CL, and ML cases have been reported in the region in the last decade [3,11,12,25]. Molecular typing of strains from canine and human hosts and from sand flies have raised doubts about the dogs’ role as main reservoirs to human infection in this region [13,14]. Moreover, studies on the blood-meals of *Ph. perfiliewi*—the most abundant sand fly species in the study area—showed a feeding preference for wild mammals (especially roe deer and European hares) and humans, while no trace of canine or rodents’ blood was observed [16]. In addition, high infection rates in *L. infantum* naturally infected sand flies were found, suggesting the existence of a dog-independent sylvatic cycle [15,16].

These findings led to the investigation of the potential role of wild mammals as natural reservoirs of *L. infantum* in the region. Different species of mammals have been found positive for *L. infantum* DNA in the study area, with an overall prevalence of 6.6% (CI 95%: 5.2–8.2), ranging among positives from 1.7% to 16.7% according to species. The overall highest positivity rates in wildlife were observed in the foothill areas of the Forlì-Cesena and Bologna provinces (Figure 1), which are the areas more affected by human leishmaniasis [19] and where a high density of sand flies has been historically reported [26,27]. Even though the results could be skewed by the non-homogeneous sample across provinces, the largest number of infected species was also detected in these provinces, perhaps as a result of higher infection pressure. The circulation of the *Leishmania* parasite in these provinces could be related to the abundance of sand flies [27]. *Ph. perfiliewi* is characterized by a patchy distribution, but it reaches higher densities in rural and semi-natural areas of the hills (hedges on the border of cultivated fields, woods, pastures) where wild animals are numerous and simple to access as blood sources.

Among the wild carnivores sampled in the present study, the highest *Leishmania* DNA detection rate was observed in wolves (16.7%; CI 95%: 3.6–41.4). This value was lower than those reported from other European countries, including other northern Italian regions, where prevalence rates of up to 50% were observed [28,29,30]. In addition, the prevalence found in foxes (4.1%; CI 95%: 2.2–6.9), ranging from 3.6% to 7.2% according to the sampling areas (Table S1), was lower than that reported previously in northwestern (12.3%), central (52.2%), and southern Italy (28.6%–40%) [30,31,32,33], and in Spain (14.1%) [34,35]. Similarly, a low *L. infantum* detection rate was found in European badgers (1.7%), when compared with the high values observed in northern Spain (26%) and northwestern Italy (53.3%) [30,36].

Among rodents, porcupines were the unique species found positive for *L. infantum* DNA with a detection rate of 1.9% (CI 95%: 0.1–9.9). To the best of our knowledge, there is only a single paper reporting porcupines of the genus *Hystrix* as a possible wild reservoir species for visceral leishmaniasis [37]. The small sample size of other species prevented a real assessment of the current prevalence in rodents in the study area. Recently, anthropophilic rodents have been found to be infected at a prevalence of 11% in plain areas of RER [38], which are historically less involved in human leishmaniasis than the foothill areas. However, the increase in seasonal abundance and the distribution of sand flies due to climate change, together with the recent detection of *Leishmania* DNA from lowland sand flies [39], indicate that the role of rodents in the epidemiology of leishmaniasis in RER needs to be monitored and further investigated.

Lagomorphs such as European hares were found infected in the study area with a detection rate of 15.4% (CI 95%: 5.9–30.6). Even though this value was lower than those reported from other Mediterranean countries, such as Spain (43.6%) and northern Greece (23.5%) [40,41], all the positive subjects (6/13, 46%) were sampled in the Bologna province (Table S1), which is the most ancient and re-emerging focus of VL in RER [11]. In addition to black rats (*Rattus rattus*) in Italy [42], infectiousness to sand flies has been demonstrated in the Iberian hare (*Lepus granatensis*), which was suggested to be the main sylvatic reservoir in the human leishmaniasis outbreak that occurred in Fuenlabrada, Spain [40,43].

Although rodents, carnivores, and lagomorphs are the orders more widely explored in leishmaniasis foci, being the most suitable wild reservoirs for the parasite [44,45], over the last decade, the list of wild animals infected with *L. infantum* has been enlarged with bats, primates, and different species of hedgehog [17,46,47,48]. *L. infantum* has been recently detected or isolated from domestic species belonging to the families Equidae and Bovidae, respectively [49,50]. However, wild artiodactyls have only been rarely investigated.

The present study provided convincing evidence for *L. infantum* DNA detection in wild boar (*Sus scrofa*) and in other two artiodactyls belonging to the family Cervidae, namely roe deer (*Capreolus capreolus*) and red deer (*Cervus elaphus*).

Moraes-Silva and colleagues [51], by combining serologic and molecular methods as well as experimental infection, concluded that domestic swine (*Sus scrofa*) is resistant to infection by *L. infantum*, excluding the possible involvement of this species in the epidemiology of visceral leishmaniasis. However, the same authors commented on a previous study [52] showing the presence of numerous amastigotes of an uncharacterized *Leishmania* species, probably *L. braziliensis*, in one pig. In the present study, wild boars showed a median Ct value of 35.9, with the lowest value above 30, probably indicating a natural resistance to the disease and a transient infection. Nevertheless, the detection of an infected animal in the spring indicates that additional research is required to clarify the role of wild boar.

Data generated in the present study suggest the need for an in-depth analysis of the role of cervids. The detection rate of Leishmania DNA in roe deer over the entire study area was 12.3% (95% CI: 8.9–16.5). Considering only the areas most affected by human leishmaniasis, it was found to be 9.4% in the province of Bologna, while the highest infection rate was detected in the province of Forlì-Cesena with 31.8% of infected roe deer. In addition, roe deer has been shown to be the preferred host species by *Ph. perfiliewi* in selected endemic sites in the RER region, according to blood-meal identification. In the same study, a *Ph. perfiliewi* specimen was found engorged with a double meal, i.e., roe deer and man, and it was infected by a high load of *L. infantum* as well [16].

Absence of pathological alterations has been a common finding among wild animals infected by *Leishmania* spp., but in a study carried out in Spain, almost half the animals tested exhibited at least one lesion compatible with leishmaniasis in their earlobe [53]. In the present study, the earlobe tissue of a single roe deer showed an ulcerated area with an inflammatory histologic pattern typical of cutaneous leishmaniasis (data not shown). Further investigation is ongoing at the time of writing this paper.

In this study, three different tissues were targeted to detect the parasite in wildlife. Earlobe samples showed a higher level of *Leishmania* DNA detection than the spleen samples. Results of lymph node examination suggested a low tendency for *L. infantum* visceralization in wildlife from RER. Other authors have also compared different tissues, with results varying between studies as well as between animal species. Abbate and colleagues [33] showed that in most foxes and wild rabbits, *L. infantum* DNA was found mainly in the spleen. In other lagomorph surveys, *L. infantum* was more frequently detected in the skin than in other tissues [29,54]. 

The mere finding of *L. infantum* DNA in a given animal species does not necessarily imply that this species is involved in *Leishmania* epidemiology, acting as a reservoir. Xenodiagnoses should be carried out to demonstrate host infectiousness to sand flies. However, it is likely that the success of host to sand fly transmission depends on the extent of skin parasite load, which has been suggested as the best marker to identify potential reservoir animal species [36,55,56]. Although the molecular analysis performed did not allow for exact quantification, the real-time PCR threshold cycle (Ct) of positivity can serve as an estimate of parasite load [15]. A low-to-intermediate number of parasites (median Ct between 25 and 35) was found in the earlobes of red deer, wolves, European hares, porcupines, roe deer, and red foxes. In the latter two species, we also found high parasite loads (Ct values < 20) in several earlobe samples. Amastigotes in skin are directly accessible to sand flies, which are known to prefer safe, hairless parts of hosts, such as the ear pinnae, and feed copiously on them [57,58].

The results of the present study showed detection of *Leishmania* DNA also in winter and spring, seasons free of adult sand flies [27]. Roe deer tested positive in all seasons, but the limited number of sampled animals does not allow for a definitive assessment to be drawn on all other species. Furthermore, the occurrence of Ct values below 25 in earlobe samples independently of the season suggests that some species could not clear the *L. infantum* infection, thus fitting the most important of the five criteria to consider a species as a reservoir of *Leishmania* parasites, i.e., the long course of infection [59,60]. In light of this, roe deer need to be further investigated, as they are among the species with low Ct values in spring, thus representing a possible host for *L. infantum* overwintering.

The present study confirmed previous findings on the existence of a sylvatic cycle for *L. infantum* in the study area. We cannot exclude that differences observed in wild hosts and parasite’s tissue tropism in RER could be linked to: (*i*) the peculiar parasitic population circulating in humans and sand flies in the region [14], being phylogenetically distant from the *L. infantum* MON-1 strains commonly affecting dogs and humans [61,62,63,64]; (*ii*) the predominant sand fly species, i.e., *Ph. perfiliewi*, in comparison with other Mediterranean endemic areas, where *Ph. pernicious* is the main vector of *L. infantum*. Finally, the exhibited multi-host infection pattern and preferential tropism for skin tissues would allow the parasite to widen its reservoir spectrum, allowing for greater exploitation of the trophic (host) activity of the vector, determined by vector host preference and host abundance. The involvement of wild animals in the parasite’s epidemiology will be determined by ongoing molecular research on the *L. infantum* DNA detected in wildlife in RER.

## 5. Conclusions

A deep knowledge of the transmission dynamics of *Leishmania* parasites is essential in order to apply control measures or monitoring programs. This study provides evidence that *L. infantum* is widespread in wildlife in RER and gives preliminary data on tissue tropism and geographic and seasonal pattern of the parasite. Additionally, the study expands the number of mammalian hosts for *L. infantum* in Italy. Further investigation is needed in order to clarify whether wildlife species should be considered maintenance hosts, sources of infection, or sentinels for *L. infantum* circulation in the re-emerging focus of leishmaniasis in Northeast Italy.

## Figures and Tables

**Table 1:** *L. infantum* real-time PCR positive wildlife species in the Emilia-Romagna region in the period 2019-2022. Detection rates (including 95% confidence interval) for the target species and tissues are reported.

Order	Species	Earlobe		Spleen		Total	
		Positive (Tested)	Prevalence (CI 95%)	Positive (Tested)	Prevalence (CI 95%)	Positive (Tested)	Prevalence (CI 95%)
Artiodactyla	Roe deer	36 (309)	11.7 (8.3–15.8)	5 (309)	1.6 (0.5–3.7)	38 (309)	12.3 (8.9–16.5)
	Red deer	1 (11)	9.1 (0.2–41.3)	0 (11)	0.0 (0.0–28.5)	1 (11)	9.1 (0.2–41.3)
	Wild boar	6 (164)	3.7 (1.4–7.8)	2 (164)	1.2 (0.2–4.3)	8 (164)	4.9 (2.1–9.4)
Carnivora	Wolf	3 (18)	16.7 (3.6–41.4)	0 (18)	0.0 (0.0–18.5)	3 (18)	16.7 (3.6–41.4)
	Red fox	13 (319)	4.1 (2.2–6.9)	2 (319)	0.6 (0.1–2.3)	13 (319)	4.1 (2.2–6.9)
	European badger	1 (59)	1.7 (0.0–9.1)	0 (59)	0.0 (0.0–6.1)	1 (59)	1.7 (0.0–9.1)
Lagomorpha	European hare	6 (39)	15.4 (5.9–30.5)	0 (39)	0.0 (0.0–9.0)	6 (39)	15.4 (5.9–30.6)
Rodentia	Porcupine	1 (54)	1.9 (0.1–9.9)	0 (54)	0.0 (0.0–6.6)	1 (54)	1.9 (0.1–9.9)
Other species ^1^		0 (104)	-	0 (104)	-	0 (104)	-
All Species		67 (1077)	6.2 (4.9–7.8)	9 (1077)	0.8 (0.4–1.6)	71 (1077)	6.6 (5.2–8.2)

^1^ species with no positive individuals, namely 62 hedgehogs, 24 fallow deer, 2 stone martens, 1 pine marten, 1 raccoon, 1 Western polecat, 7 squirrels, 5 dormices, 1 common dormouse.

**Table 2:** Lymph node analysis on 23 animals already tested positive for *L. infantum* DNA in the earlobe and/or spleen.

Order	Species	No. of Lymph Node Positive Animals (No. Tested)			
		Earlobe	Spleen	Both	Total
Artiodactyla	Roe deer	2 (10)	0 (1)	1 (2)	3 (13)
	Red deer	1 (1)			1 (1)
	Wild boar	0 (1)			0 (1)
Carnivora	Wolf	1 (3)			1 (3)
	Red fox	1 (3)		0 (1)	1 (4)
	European badger	0 (1)			0 (1)
	total	5 (19)	0 (1)	1 (3)	6 (23)

**Table 3:** Overall prevalence (including 95% confidence interval) of *L. infantum* DNA in wildlife in the provinces of the Emilia-Romagna region.

Province	No. of Positive (Tested Animals)	Prevalence (%)	CI 95%
Bologna	27 (309)	8.7	(5.8–12.5)
Forlì-Cesena	22 (188)	11.7	(7.5–17.2)
Modena	11 (250)	4.4	(2.2–7.7)
Ravenna	3 (40)	7.5	(1.6–20.4)
Reggio Emilia	1 (162)	0.6	(0.0–3.4)
Rimini	7 (128)	5.5	(2.2–10.9)

CI: confidence interval.

**Table 4:** Prevalence (including 95% confidence interval) of *L. infantum* real-rime PCR positive species along the weather seasons.

		Season											
Order	Species	Spring			Summer			Autumn			Winter		
		N° Positive (n° Tested)	Prevalence	95% CI	N° Positive (n° Tested)	Prevalence	95% CI	N° Positive (n° Tested)	Prevalence	95% CI	N° Positive (n° Tested)	Prevalence	95% CI
Artiodactyla	Red deer							1 (6)	16.7	(0.4–64.1)	0 (5)	0	(0–52.2)
	Roe deer	1 (75)	1.3	(0–7.2)	6 (53)	11.3	(4.3–23)	13 (64)	20.3	(11.3–32.2)	18 (117)	15.4	(9.4–23.2)
	Wild boar	1 (10)	10	(0.3–44.5)	0 (21)	0	(0–16.1)	3 (77)	3.9	(0.8–11)	4 (56)	7.1	(2–17.3)
Carnivora	European badger	1 (6)	16.7	(0.4–64.1)	0 (10)	0	(0–30.8)	0 (21)	0	(0–16.1)	0 (22)	0	(0–15.4)
	Red fox	0 (4)	0	(0–60.2)	5 (157)	3.2	(1–7.3)	4 (64)	6.3	(1.7–15.2)	4 (94)	4.3	(1.2–10.5)
	Wolf				0 (1)	0	(0–97.5)	0 (6)	0	(0–45.9)	3 (11)	27.3	(6–61)
Lagomorpha	European hare	0 (7)	0	(0–41)	0 (4)	0	(0–60.2)	1 (16)	6.3	(0.2–30.2)	5 (12)	41.7	(15.2–72.3)
	Porcupine	0 (8)	0	(0–36.9)	0 (13)	0	(0–24.7)	0 (17)	0	(0–19.5)	1 (16)	6.3	(0.2–30.2)
All species		3 (113)	2.7	(0.6–7.6)	11 (288)	3.8	(1.9–6.7)	22 (329)	6.7	(4.2–10.0)	35 (347)	10.1	(7.1–13.8)

CI: confidence interval.

## Data Availability

Data generated or analyzed during this study are included in the published article.

## Supplementary Materials

The following supporting information can be downloaded at: https://www.mdpi.com/article/10.3390/pathogens11111308/s1, Table S1: Prevalence (including 95% confidence interval) of *L. infantum* real-rime PCR positive species among the provinces of the study area.
